# Scrotal abscess caused by *Candida glabrata* in a patient with uncontrolled diabetes mellitus and chronic renal failure

**DOI:** 10.1590/S1678-9946202466071

**Published:** 2024-12-06

**Authors:** Mehmet Ali Tüz, Derya Tuna Ecer, Tuğba Kula Atik, Oktay Yapıcı

**Affiliations:** 1 Balikesir University, Medical Faculty, Infectious Diseases and Clinical Microbiology Department, Balikesir, Turkey; 2 Balikesir University, Medical Faculty, Medical Microbiology Department Balikesir, Turkey

**Keywords:** Candida, Glabrata, Scrotal abscess, Candidemia

## Abstract

*Candida glabrata* is a yeast which incidence has increased in recent years and usually causes urogenital and bloodstream infections. Its resistance to fluconazole hinders *C. glabrata* infections treatment. This case report presents a case of candidemia and scrotal abscess caused by *C. glabrata*, which was successfully treated with liposomal amphotericin B. The primary treatment options for *C. glabrata* candidemia are echinocandins and amphotericin B formulations. However, echinocandins and lipid-based amphotericin B formulations do not properly pass through the urinary system. Amphotericin B deoxycholate has a high risk of side effects and is difficult to obtain. The treatment option for candidemia caused by fluconazole-resistant *C. glabrata* secondary to urinary tract infection is unclear and there are no sufficient studies. For treatment, liposomal amphotericin B may be considered, especially for scrotal and prostatic fluconazole-resistant fungal abscesses. More studies comparing the penetration of antifungals into scrotal and prostatic tissue and the success of antifungal treatment in these tissue infections are needed.

## INTRODUCTION

Candidemia indicates the presence of *Candida* species in the blood. Although *Candida albicans* is the most common species, other *Candida* species have been isolated with growing cases in recent years^1^. *Candida glabrata (Nakaseomyces glabrata)* is a yeast that can be found in the normal microbiota of healthy individuals and can cause various infections in humans^2^. Mucosal and systemic infections caused by *C. glabrata* have raised significantly due to the increase in the number of patients with immunocompromised systems and the increased clinical use of various antifungal drugs. Accordingly, in many health centers, infections caused by *C. glabrata* have begun to take the second or third place after *C. albicans* infections^3^.

Generally, *C. glabrata* can cause bloodstream or urogenital infections and sometimes infect other areas. Invasive candidiasis is defined as the hematogenous spread to more than one internal organ (e.g., eye, kidney, heart valves, brain). Clinical signs on physical examination that indicate possible hematogenous spread of *Candida* spp. include characteristic ocular lesions, skin lesions, and, much less commonly, muscle abscesses^1,4^. Positive blood cultures stand as the gold standard for the diagnosis of invasive candidiasis and candidemia. Blood cultures should be obtained from all patients with suspected candidemia. In patients with dissemination to others organs (e.g., skin lesions or parenchymal involvement), a biopsy should be conducted for both microbiological and histopathological evaluation. Non-culture-based methods (beta-D-glucan, etc.) may be used to aid in diagnosis of invasive candida infections when blood cultures are negative^5^.

Treatment of candidemia generally consists of monotherapy; the benefit of combination therapy has not been established. Echinocandins, azoles and amphotericin B formulations may be used. Initial treatment with echinocandins is recommended for patients with non-neutropenic candidemia. In patients who are not neutropenic or critically ill, fluconazole may also be used as an alternative agent if fluconazole resistance has not been reported. Liposomal amphotericin B is preferred to treat candidemias in which resistance to echinocandins and azoles is suspected or proven (infection caused by *C. glabrata, C. krusei*)^1,6^.

This report discusses a case of *C. glabrata* candidemia and associated scrotal abscess, treated with liposomal amphotericin B and discharged in good health.

## CASE REPORT

A 67-year-old male patient, diagnosed with diabetes mellitus, hypertension, chronic renal failure and a history of bypass surgery, had many hospital admissions due to complaints of cold, tremors, weakness and weight loss that had been going on for two months. Notably, 20 days before the last admission, he was hospitalized in the internal medicine department with preliminary diagnoses of gastroenteritis and acute renal failure and received intravenous (IV) antibiotics for four days. On the second day of hospitalization, urine culture and two sets of blood cultures were taken due to high fever. The blood culture was collected on the BD BACTEC FX (Becton Dickinson, USA) automated blood culture system. Urine culture presented >100,000 CFU/ml *Candida* spp. Initially, 1×200 mg of fluconazole was prescribed, followed by 1×100 mg IV maintenance was added to the treatment. Before the blood culture results, cefixime 1×400 mg and fluconazole 1×100 mg tablets were prescribed and he was discharged with a urinary catheter.

Two weeks later, he returned to the emergency ward complaining of cold, tremors and weakness. Physical examination indicated 38.3 °C, other vital signs were stable, suprapubic tenderness, and slight swelling around the right testicle. The patient stated that his catheter was removed a few days ago, but was reinserted due to globe vesicle. Laboratory testing yielded the following counts: white blood cells (WBC) 20.4×10^3^ µL (87.1% PMNL); C-reactive protein (CRP) 158 mg/dL, Hgb 9.3 g/dL; procalcitonin 0.2 ng/mL; and creatinine 1.86 mg/dL (GFR:36 mL/min/1.73 m^2^) was present. *C. glabrata* growth was observed in two sets of blood cultures taken during his hospitalization 20 days before. The patient was admitted to the infectious diseases service for further investigation and treatment with the preliminary diagnosis of candidemia. After three sets of blood cultures and urine cultures were taken, liposomal amphotericin B was started at a dose of 5 mg/kg/day.

When the patient's medical history was questioned in detail after his hospitalization, it was found that he had been diagnosed with chronic pancreatitis one year ago because of fatigue, diarrhea and abdominal pain, and that he had not achieved adequate glycemic control for a long time. During hospitalization in internal medicine department HbA1c measurement was 100 mmol (11.3%). Although diabetes treatment was arranged during the hospitalization 10 days before, hyperglycemic and hypoglycemic episodes were observed during day-time follow-up in the wards.

Ophthalmological examination yielded no pathological findings, similar to transthoracic echocardiography, which showed no vegetation. Endocrinological recommendations for glycemic control were followed.

**Figure 1:**
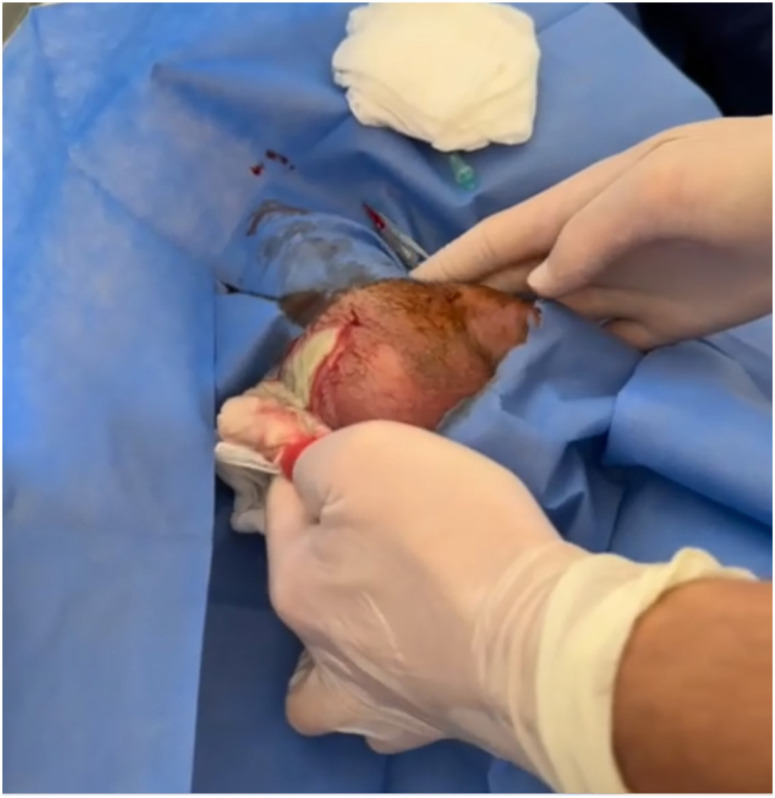
Scrotal abscess percutaneous drainage caused by *C. glabrata*.

During follow-up, scrotal ultrasound performed due to increasing testicular swelling and newly developed local heat revealed a 6-cm diameter collection containing moving echogenicity and air images, which was interpreted as a scrotal abscess. Percutaneous abscess drainage was performed by the urologist and a drain was placed (Figure 1). Abscess aspirate material was sent for culture and meropenem 3×1 gr IV was added to the treatment. Laboratory testing yielded the following information: WBC 24.7×10^3^ µL (88.6% PMNL); CRP 172 mg/dL; procalcitonin 0.5 ng/mL; creatinine 1;65 mg/dl (GFR: 42 mL/min/1.73 m^2^).

As Gram-positive cocci were growing in the abscess culture on the 4^th^ day of follow-up, teicoplanin was added to the treatment at a 1×600 mg IV maintenance dose after the first three doses, 12 h apart. During follow-up, the dose of antibiotics was revised according to creatinine clearance.

On the 7^th^ day of follow-up, *C. glabrata* with fluconazole-susceptible dose dependent and ampicillin-sensitive *Enterococcus faecalis* grew in the abscess culture, but there was no growth in blood cultures taken at admission or at other days. Broth microdilution methods were used to test the susceptibility of *C. glabrata* to fluconazole. The patient's urine culture on admission grew >100,000 CFU/mL *C. glabrata*, and there was no growth in urine cultures taken after the second day of treatment. High fever did not recur after the 2^nd^ day of follow-up. On the 7^th^ day of follow-up laboratory findings were as follows: WBC 12.8×10^3^ µL (77.7% PMNL); CRP 37 mg/dl; creatinine: 1.46 mg/dL were discontinued, ampicillin sulbactam 4×2 g IV was started, and liposomal amphotericin B treatment was continued, considering that appropriate high-dose treatment could not be provided with fluconazole, since the GFR ranged 40–55 during follow-up.

**Figure 2:**
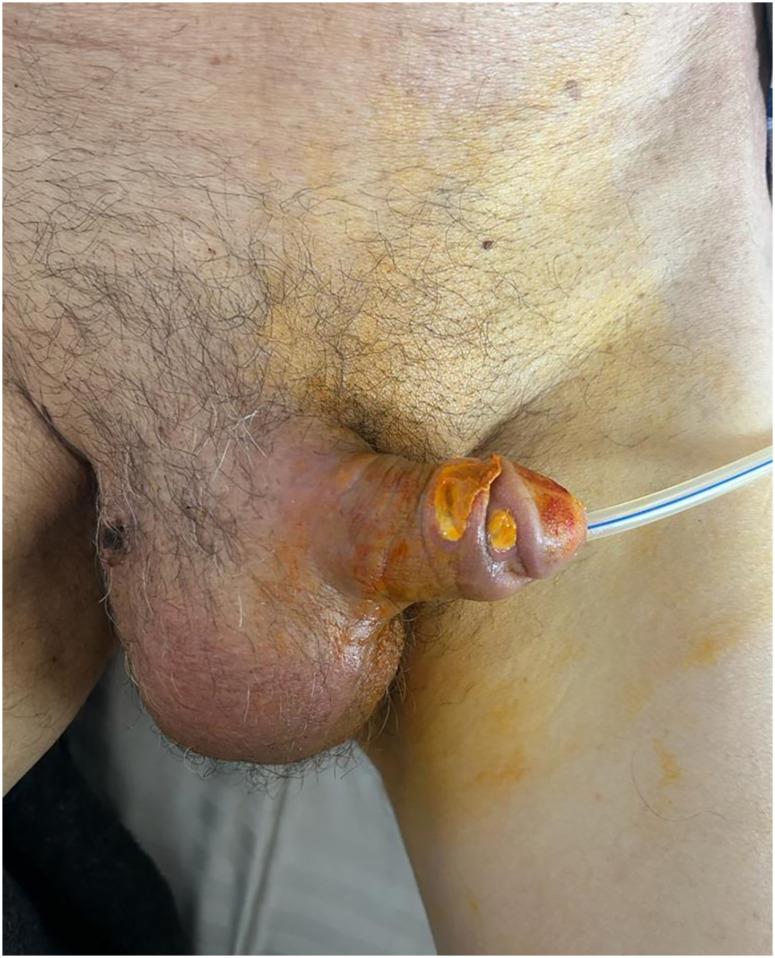
Scrotal view after drain removal before discharge.

On the 11^th^ day of follow-up, the following counts were taken: WBC 12.7×10^3^ µL (69.9% PMNL); CRP 18.1 mg/dL; procalcitonin 0.2 ng/ml; creatinine 1.49 mg/dl (GFR: 47 mL/min/1.73 m^2^). By analyzing the control ultrasound, it was verified that the abscess size had decreased; the drain flow also decreased. The patient was evaluated by urology and the percutaneous drain was removed (Figure 2). Lesions with purulent discharge on the penis that developed during follow-up were considered to be fistulization of the abscess and were treated with daily debridement and dressings. Ampicillin-sulbactam and amphotericin B therapies were suspended on the 10^th^ and 17^th^ day, respectively. The last laboratory testing yielded the following values: WBC was 10.6×10^3^ µL; CRP 7 mg/dL; creatinine 1.40 mg/dL (GFR 51 mL/min/1.73 m^2^). The patient's catheter was removed and his clinical outcomes improved significantly. He was then discharged with the prescription of amoxicillin-clavulanate 2×1000 mg and fluconazole 1×400 mg tablet. One week later, at the urological follow-up, it was observed that the abscess had completely regressed and the clinical findings were resolved.

## DISCUSSION

Echinocandins or amphotericin B are usually the first-line treatment for infections caused by *C. glabrata*—especially candidemia and invasive candidiasis^1^. Due to the delayed killing kinetics of amphotericin B against C. *glabrata in vitro*, echinocandins are more prominent in candidemia caused by *C. glabrata* ^7^. However, some studies have shown increased echinocandin resistance in *C. glabrata* bloodstream isolates^8^. In the presence of candidemia with moderate-fluconazole-sensitive (susceptible, increased exposure) *C. glabrata* strains, 5-7 days after IV echinocandin or amphotericin B treatment, oral fluconazole maintenance treatment can be started for cases presenting no growth in control blood cultures^1^. The European Society for Clinical Microbiology and Infectious Diseases (EUCAST) recommends that isolates with MICs below 16 mg/L in *C. glabrata* infections should be considered as dose-dependent susceptible and only appropriate high-dose treatment is recommended^9^.

Treatment of candidemia caused by *C. glabrata* that develops secondary to a urinary tract infection is more difficult because most antifungal treatments do not pass adequately into the urine^1,10^. Echinocandins and lipid formulations of amphotericin B do not pass well through the urine. For fluconazole-resistant *C. glabrata* urinary infections, amphotericin B deoxycholate can be used intravenously and/or by bladder irrigation, but the possibility of side effects is higher and it is difficult to acquire in Turkey^1,10^. In urinary infections with fluconazole-sensitive strains, successful treatment can be achieved with high-dose fluconazole, especially in cases of candidemia co-infection. However, in co-infection cases, it is known that failure to achieve adequate blood and tissue concentrations of fluconazole is the most important determinant of complications and mortality^10,11^. In our case, empirical liposomal amphotericin B was started because of the history of fluconazole use and the growth of *C. glabrata* in previous cultures. After the culture and susceptibility results were obtained — although he was infected with a fluconazole-susceptible dose dependent (susceptible, increased exposure) strain — due to the diagnosis of chronic renal failure and the variable glomerular filtration rate during follow-up, fluconazole was not interrupted because of the risk of not achieving adequate blood and tissue concentrations, and treatment with liposomal amphotericin B was continued. On the other hand, although there was growth in the urine culture on first admission, there was no growth in the urine cultures taken after the 48^th^ hour of treatment.

Although echinocandins and amphotericin B are indicated among the treatment options for abscesses caused by *C. glabrata* that may be associated with a urinary tract infection — such as a prostate abscess — there are insufficient studies on antifungal concentrations in tissues such as the prostate and scrotum and antifungal treatment options for abscesses in these regions^1,11^. In prostate abscesses, a 4-week course of antifungal treatment seems to be sufficient^11^. In our case, the total duration of antifungal treatment was approximately four weeks.

Uncontrolled diabetes is one of the most important risk factors for the development of candidal abscesses in the urinary system and candidemia^1,10,11^. In our case, uncontrolled diabetes due to chronic pancreatitis was thought to be a facilitating factor for candida infection.

## CONCLUSION

The treatment option for candidemia caused by fluconazole-resistant *C. glabrata* secondary to urinary tract infection is unclear and there are no sufficient studies. Liposomal amphotericin B may be considered, especially for scrotal and prostatic fluconazole-resistant fungal abscesses. More studies are needed comparing the penetration of antifungals into scrotal and prostatic tissue and the success of antifungal treatment in these tissue infections.
